# Swallowing the pill of adverse effects: A qualitative study of patients' and pharmacists' experiences and decision‐making regarding the adverse effects of chronic pain medications

**DOI:** 10.1111/hex.13399

**Published:** 2021-12-21

**Authors:** Lise Dassieu, Emilie Paul‐Savoie, Élise Develay, Ana Cecilia Villela Guilhon, Anaïs Lacasse, Line Guénette, Kadija Perreault, Hélène Beaudry, Laurent Dupuis, the Quebec Consortium on Adverse Effects of Pain Medications

**Affiliations:** ^1^ Research Center of the Centre hospitalier de l'Université de Montréal Montreal Quebec Canada; ^2^ Department of Biomedical Sciences Faculty of Medicine, Université de Montréal Montreal Quebec Canada; ^3^ Quebec Pain Research Network Sherbrooke Quebec Canada; ^4^ School of Nursing, Faculty of Medicine and Health Sciences Université de Sherbrooke Longueuil Quebec Canada; ^5^ Department of Community Health Sciences Faculty of Medicine and Health Sciences Université de Sherbrooke Longueuil Quebec Canada; ^6^ Department of Health Sciences Université du Québec en Abitibi Témiscamingue Rouyn‐Noranda Quebec Canada; ^7^ Faculty of Pharmacy Université Laval Quebec City Quebec Canada; ^8^ Centre interdisciplinaire de recherche en réadaptation et en intégration sociale (CIRRIS) Centre intégré universitaire de santé et de services sociaux de la Capitale‐Nationale Quebec City Quebec Canada; ^9^ Department of Rehabilitation, Faculty of Medicine Université Laval Quebec City Quebec Canada

**Keywords:** adverse effects, analgesics, chronic pain, community pharmacists, decision‐making, person‐centred care, qualitative research

## Abstract

**Introduction:**

Pharmacological treatments of chronic pain can lead to numerous and sometimes serious adverse effects. Drawing on a social science approach to chronic illness, this study aimed to understand the experiences of people living with chronic pain and community pharmacists regarding the definition, prevention and management of analgesic adverse effects.

**Methods:**

This qualitative study proceeded through 12 online focus groups (FGs) with people living with chronic pain (*n* = 26) and community pharmacists (*n* = 19), conducted between July 2020 and February 2021 in the province of Quebec, Canada. The semistructured discussion guides covered participants' definitions of adverse effects and decision‐making regarding their prevention and management. Discussions were audio‐recorded, transcribed verbatim and analysed using grounded theory.

**Results:**

Both people with chronic pain and pharmacists provided varying definitions of analgesic adverse effects depending on patients' social and clinical characteristics. Present quality of life and serious long‐term risks related to treatment were described as key dimensions influencing adverse effect appraisal. Dilemmas and discrepancies occurred between patients and pharmacists when choosing to prioritize pain relief or adverse effect prevention. Some patients lacked information about their medications and wanted to be more involved in decisions, while many pharmacists were concerned by patients' self‐management of adverse effects. Preventing opioid‐related overdoses often led pharmacists to policing practices. Despite most pharmacists wishing they could have a key role in the management of pain and adverse effects face organizational and financial barriers.

**Conclusion:**

Defining, preventing and managing adverse effects in the treatment of chronic pain requires a person‐centred approach and shared decision‐making. Clinical training improvements and healthcare organization changes are needed to support pharmacists in providing patients with community‐based follow‐up and reliable information about the adverse effects of chronic pain treatments.

**Patient or Public Contribution:**

A person with lived experience of chronic pain was involved as a coinvestigator in the study. He contributed to shaping the study design and objectives, including major methodological decisions such as the choice of pharmacists as the most appropriate professionals to investigate. In addition, 26 individuals with chronic pain shared their experiences extensively during the FGs.

## 1 INTRODUCTION

### 1.1 Background

Chronic pain is a major public health issue affecting one in five adults in North America^1^, ^2^, ^3^ and leading to significant negative impacts on patients' daily living.^3^, ^4^ Pharmacological therapies are prescribed to a majority of patients (62%–84%)^5^, ^6^, ^7^ as part of the recommended multidisciplinary biopsychosocial approach to chronic pain treatment.^8^, ^9^ In North America, the most commonly prescribed medications to manage chronic pain are opioids, nonsteroidal anti‐inflammatory drugs (NSAIDs), acetaminophen, antidepressants, anticonvulsants and muscle relaxants.^5^, ^10^, ^11^, ^12^

Chronic pain pharmacotherapies are highly beneficial for patients in terms of pain relief and quality of life. However, most analgesics can lead to adverse effects, which may intensify when several prescribed and/or over‐the‐counter medications are combined.^13^, ^14^ For example, commonly prescribed medications to treat chronic pain, such as opioids^15^ and antidepressants,^16^ frequently cause nausea, dry mouth, drowsiness, constipation, dizziness and/or headache.^15^, ^16^ Furthermore, clinically serious adverse effects of long‐term opioid therapies, such as opioid use disorder and overdose‐related respiratory arrest, have been widely described in the past few years.^17^, ^18^, ^19^ NSAIDs, for their part, increase the risks for serious cardiovascular and renal diseases.^20^, ^21^, ^22^ In addition to being detrimental to patients' physical health condition, adverse effects often lead to negative consequences on quality of life^11^, ^23^ and can deteriorate the psychological condition.^24^ Preventing and managing adverse effects of analgesics and associated risks can thus lead to significant challenges for both patients and clinicians, especially for pharmacists, who play an essential role in treatment optimization as part of a primary care approach to chronic pain.^25^, ^26^, ^27^ In several Canadian jurisdictions, pharmacists' scope of practice has been recently extended. They can now adapt or manage prescriptions and independently prescribe medications under certain conditions.^26^, ^28^ Therefore, they are more than ever at the front line to monitor, prevent and manage adverse effects of chronic pain pharmacotherapies.

Available data on analgesic adverse effects come primarily from randomized clinical trials, which makes these data difficult to transfer to real‐life experiences.^29^ Though the nature and frequency of these adverse effects have been examined in experimental settings, little is known on the experiences and concerns of people living with chronic pain and pharmacists regarding adverse effects and their management. Indeed, the most frequent or clinically serious adverse effects may not be the most problematic in the daily experience of patients and pharmacists. The few studies examining patient and clinician perspectives regarding chronic pain pharmacotherapies focused on opioids only and did not include pharmacists.^30^, ^31^, ^32^, ^33^ One qualitative study underscored that patients and physicians had distinct preferences regarding the management of opioid‐induced constipation.^30^ Furthermore, several studies highlighted shared concerns between patients and physicians regarding risks of serious adverse effects, such as opioid use disorder and overdose, especially in the context of the ongoing overdose epidemic.^32^, ^34^, ^35^, ^36^, ^37^, ^38^, ^39^ However, studies showed that divergent perspectives on opioid tapering were challenging for patient–provider communication and could result in increased patient stigmatization as well as negative impacts on pain outcomes.^38^, ^40^, ^41^, ^42^ Currently, there is no research examining the experience and management of the adverse effects of diverse chronic pain pharmacotherapies from the double perspective of patients and pharmacists. Such research is highly needed to understand patients' and pharmacists' support needs in the daily management of pain and drug adverse effects.

### 1.2 Objectives and theoretical framework

The objective of this study was to understand the experiences and decision‐making of people living with chronic pain and community pharmacists regarding the adverse effects of analgesics. More precisely, the study sought to understand: (a) how, and according to which criteria, people with chronic pain and pharmacists define and classify the adverse effects of pain medications; and (b) how people with chronic pain and pharmacists make decisions to prevent and manage the adverse effects of pain medications in their daily life or practice.

The concept of adverse effects used in this study included any unfavourable, unpleasant or harmful outcome following the intake of an analgesic drug or the interaction between an analgesic drug and other medications.^43^, ^44^ Adverse effects are distinct from the broader concept of side effects referring to all outcomes that do not pertain to the drug's primary therapeutical goal, be they considered adverse, neutral or beneficial.^43^

This study was based on a qualitative approach to the social experience of chronic illness and the provider/patient relationship, as theorized by interactionist health sociology.^45^, ^46^, ^47^, ^48^, ^49^ More precisely, this study was inspired by the theoretical framework of the ‘meaning of medication' defined by Conrad.^50^ This social‐constructionist approach proposes an alternative analysis of adherence by focusing on patients' daily experience of chronic medication intake. It offers a person‐centred perspective on the management of chronic illness and patients' adaptive strategies to integrate the treatment into their daily living.^46^, ^50^ According to Conrad, it is essential to analyse patients' diverse medication usages independently from their conformity to medical prescription, to understand patients' strategies for living with—or in spite of—their adverse effects.^50^ Such an approach enabled us to examine the criteria people with chronic pain utilized to classify the effects of their medication as adverse effects, and the strategies they deployed to manage these adverse effects, including treatment modifications. This theoretical framework also supported our understanding of pharmacists' experiences through the analysis of how the management of analgesic adverse effects was included in their daily practice and influenced by interactions with their patients through their convergent and divergent views. Such a symmetrical approach to patients' and pharmacists' experiences intends to open avenues for improving communication in the clinical relationship to optimize the management of adverse effects.

## 2 METHODS

The methods are reported following the Standards for Reporting Qualitative Research (SRQR) recommendations.^51^

### 2.1 Design and recruitment

This was a qualitative study using online (Zoom™) focus groups (FGs) with people living with chronic pain and community pharmacists. Data were collected in the province of Quebec, Canada, between July 2020 and February 2021. The research team conducted six FGs with people living with chronic pain and six FGs with pharmacists. Each FG comprised three to five participants, as recommended in the literature for online FGs.^52^, ^53^ The study was approved by the Research Ethics Boards of the *Centre hospitalier de l'Université de Montréal* and the *Université du Québec en Abitibi‐Témiscamingue*. All participants provided written informed consent by email and all data were anonymized.

**Figure 1:**
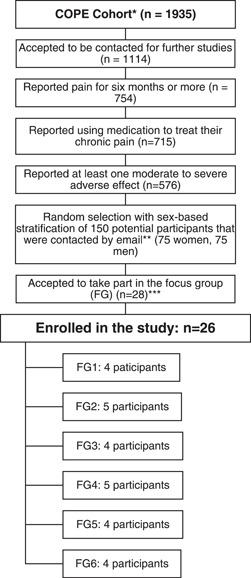
Recruitment procedure for participants living with chronic pain. *The Chronic Pain Treatment (COPE) cohort study included individuals meeting the following eligibility criteria: (1) reporting persistent or recurrent pain for more than 3 months; (2) being at least 18 years of age; (3) being able to complete a web‐based questionnaire in French and (4) living in the province of Quebec. **The email offering to participate in the focus group was sent to 50 cohort participants in June 2020, 50 cohort participants in July 2020, and 50 cohort participants in August 2020. ***Two participants expressed their interest to participate but were not enrolled because they were unavailable on the scheduled date for the last focus group (FG)

Participants living with chronic pain were recruited in a provincial web‐based prospective cohort study examining pharmacological and nonpharmacological treatments used by adults living with chronic pain.^54^ Additional information about the cohort study and recruitment procedures is shown in Figure 1. For this qualitative study, eligibility criteria included: (a) living with pain for at least 6 months, (b) using pharmacological pain treatments, and (c) experiencing one or more moderate to severe adverse effects. Among those consenting to be contacted for other studies, 150 eligible individuals were randomly selected with sex‐based stratification. They were sent an email offering them to participate in an FG. The study coordinator scheduled appointments with those who answered positively until all FGs were complete. A total of 26 people with chronic pain were recruited (Figure 1).

As FG guidelines recommend internal homogeneity of participants,^55^ the study coordinator scheduled distinct groups for participants who used opioid treatments and those using nonopioid treatments. Indeed, as several studies highlighted the strong stigma surrounding opioids,^39^, ^56^, ^57^ it was essential to ensure that the participants would be comfortable talking about their treatment without worrying about being judged by their peers. Participants were asked which types of treatments they were using during the recruitment, to include them in the appropriate group. Three FGs were conducted with people using opioid treatments and three with people using nonopioid medications.

To be eligible, pharmacists had to work in community pharmacies and/or family medicine groups in the province of Quebec. Family medicine groups are multidisciplinary primary care facilities offering services by general practitioners, pharmacists, nurses and/or social workers.^58^

Pharmacists were recruited using diverse strategies, including announcements in professional associations' newsletters, websites and/or social media pages, invitations shared in the researchers' professional network and the snowball method. Among the 20 pharmacists who accepted to participate, 19 were finally included (one cancelled participation due to insufficient time). Sociodemographic characteristics of participants are described in Tables 1 and 2.

**Table 1:** Participants' characteristics: Individuals living with chronic pain

Characteristic	*N*
Total number of participants with chronic pain	26
Age	
30–39	1
40–49	6
50–59	6
60–69	10
≥70	3
Gender	
Female	12
Male	14
Ethnicity	
White	24
Indigenous/First Nations	2
Average pain intensity in the last 2 weeks	
Mild (1–3/10)	2
Moderate (4–6/10)	17
Severe (7–10/10)	7
Pain duration (years)	
1–10	2
11–20	8
21–30	8
31–40	4
>40	4
Origin(s) of pain^a^	
Accident	7
Disease	4
Trauma/repeated movement	5
Pregnancy/childbirth	2
Undetermined	9
Pain location(s)^a^	
Multisite/generalized	14
Head/face	13
Neck	17
Shoulder	16
Arm/elbow/wrist	14
Hand	11
Back	24
Chest/rib	5
Abdomen	6
Hip	13
Buttock/genitals	8
Leg/ankle	15
Knee	15
Foot	15
Provider(s) prescribing pain medication^a^	
Family physician	16
Multidisciplinary pain treatment clinic	7
Other specialist	5
Work status	
Employed	6
Unemployed	4
Invalidity pension/paid sick leave	7
Sick leave without financial compensation	2
Retired	7
Annual income	
<CDN $20,000	11
CDN $20,000 to CDN $40,000	5
CDN $40,000 to CDN $60,000	5
CDN $60,000 to CDN $80,000	3
>CDN $80,000	2

^a^ Multiple responses accepted.

**Table 2:** Participants' characteristics: Pharmacists

Characteristic	*N*
Total number of pharmacists	19
Age	
25–29	7
30–34	7
35–39	4
55–59	1
Gender	
Female	11
Male	8
Ethnicity	
White	16
Asian	2
Black	1
Type of practice	
Community pharmacy only	8
Community pharmacy and family medicine group	11
Years of practice	
0–4	6
5–9	8
≥10	5

### 2.2 Data collection

FGs lasted between 90 and 120 min, with a 10‐min break for participants' comfort. FGs were conducted by two experienced research assistants with backgrounds in psychology and sociology. One of them moderated the discussion while the other one observed, took notes and assisted participants with technical issues when needed. Each participant was given a pseudonym to protect anonymity during discussions. FGs were audio‐recorded and transcribed verbatim. Data collection was supervised by the study's principal investigators. After the FGs, participants completed an online sociodemographic questionnaire, and they received CDN $75 for their time.

The semistructured discussion guides included open‐ended questions pertaining to participants' definition and management of adverse effects (see Supporting Information Material: discussion guides for patients and pharmacists). The moderators used prompts to help participants develop their narratives and make sure all of them had a chance to talk. The moderators did not establish a preliminary list of adverse effects, allowing participants to decide which effects they wanted to include in this category.

### 2.3 Data analysis

Data analysis was driven by Corbin and Strauss' grounded theory methodology.^59^ The analysts went back and forth between data collection and analysis to be the closest possible to theoretical saturation. The research team used several strategies to assess and foster saturation throughout the study. First, after each FG, both moderators wrote detailed reports documenting both surprising/new and redundant elements discussed by participants. Furthermore, the analysts started analysing the data concurrently with data collection. Team meetings after each FG enabled them to adapt prompts in the forthcoming FGs to verify analytic ideas and to explore unanticipated topics emerging from participants' discussions (e.g., adverse effects of natural products and cannabis). In addition, the initial research protocol included a possibility for conducting additional one‐on‐one interviews with new participants in the case 12 FGs would not provide sufficient data. We finally did not use this strategy as we could develop a consistent analysis using the data from the FGs.

In this study, the analysts adopted an interpretivist epistemological positioning considering that analytic categories or themes are coherent constructs resulting from the researchers' interpretations of participants' narratives.^59^, ^60^ Three members of the research team were involved in data analysis and code development. The involvement of three analysts coming from various disciplinary and professional backgrounds (social sciences, community health, clinical psychology) offered diversified insights into the data, fostering reflexivity and enriching interpretations.

The analysts used an iterative and reflexive approach combining attention to the research objectives with their inductive interpretations of data. We used Corbin and Strauss' constant comparative method with open and axial coding strategies to develop both a comprehensive understanding of each FG and a comparative perspective on the data.^59^ NVivo‐12 Software^61^ supported data storage and management. Memo writing throughout the analysis process helped us to progressively build the analytic categories. Regular team discussions supported the constant comparison procedures and allowed us to produce an integrated interpretation of the data set. Given that participants were francophones, a professional translator translated all cited quotes and the discussion guides for this article. The translation was double‐checked by the research team.

## 3 RESULTS

**Figure 2:**
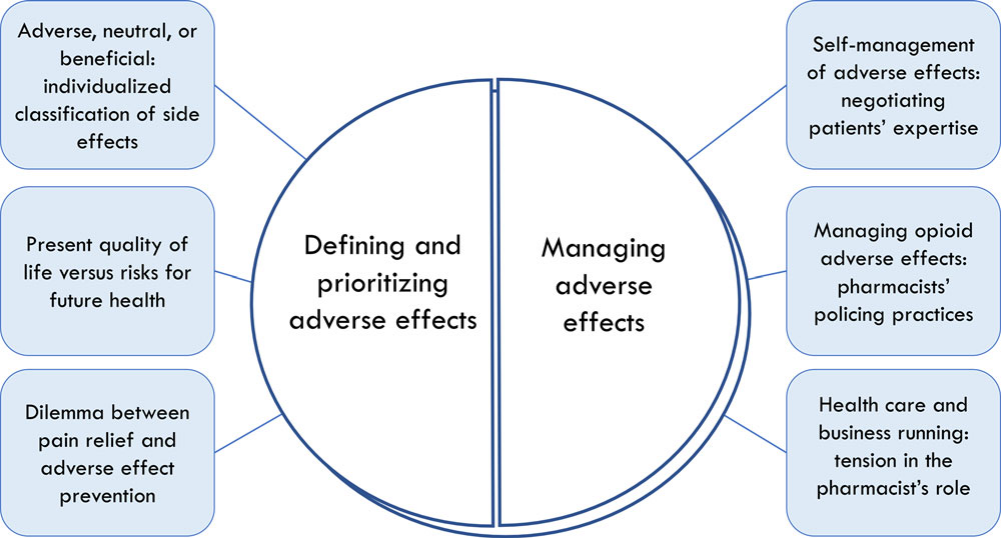
Swallowing the pill of analgesic adverse effects

This section centres on (1) patients' and pharmacists' experiences and dilemmas while defining, classifying and prioritizing analgesic adverse effects; (2) patients' and pharmacists' practices, expectations and challenges in the management of analgesic adverse effects. A graphical representation of the analytical categories and their dimensions is provided in Figure 2.

### 3.1 Defining and prioritizing adverse effects: An individualized benefit‐risk balance

#### 3.1.1 Adverse, neutral or beneficial: Individualized classifications of side effects

People with chronic pain and pharmacists defined and prioritized adverse effects according to their perspective on the benefit/risk balance of pain medication intake. Participants actively constructed the notion of ‘adverse effect’ through the classification of the different effects as either undesirable/adverse, tolerable or desirable. For both patients and pharmacists, this classification resulted from their individual assessment of medication benefits and risks, which varied according to diverse characteristics, such as the patient's age, comorbidities, professional activity and familial situation. The importance of personalizing the treatment was recurrent in FG discussions. Depending on the patient's situation, a given effect could be alternatively defined as an adverse, undesirable effect or as a desirable, helpful effect. For example, some pharmacists explained that drowsiness was a positive side effect for patients suffering from insomnia, a minor adverse effect for retired patients, while it was concerning for patients with a professional activity requiring productivity or driving:

Sometimes, I recommend a pain medication and I say, ‘It's going to make you drowsy’. Some patients say, ‘No problem. I'm retired. I'm home all day’. But some others who have to work, who are on the road, truckers, for them it's just impossible. So, their occupation and schedule can make a difference when choosing a treatment. (Pharmacist, Part.38‐FG10)

According to several patients, some side effects of their pain medication positively impacted their other health conditions:

I have fibromyalgia, severe generalized arthritis and emphysema, and the medication allows me to be active. I can go for a walk, I can concentrate enough to read a book, which I wasn't able to do before because of my ADHD [Attention Deficit/Hyperactivity Disorder]. (Patient, Part.7‐FG2)

#### 3.1.2 Present quality of life versus risks for future health

Participants appraised medications' benefit/risk balance through two temporalities: present quality of life and risks for future health. Participants hierarchized adverse effects according to the importance they gave to each of these temporalities in the process of finding an appropriate benefit/risk balance.

The impact of adverse effects on their present quality of life was an essential concern reported by most patients. Analgesic adverse effects often affected patients' social, professional and personal living conditions. Patients regretted that some adverse effects interfered with their activities as much as the pain itself. Adverse effects affecting cognitive functions (e.g., memory loss, brain fog), physical aptitudes (e.g., tremors, fatigue) or self‐esteem (e.g., weight gain) were particularly concerning for patients. Several patients were concerned that these adverse effects could be detrimental to their social and familial relationships:

One of the side effects is that it completely throws off your sleep cycle, so you no longer keep the same hours as everyone else. That complicates your social life and your family life, because you're totally out of the loop, you can't keep up with the others. (Patient, Part.8‐FG2)

Some pharmacists reported being committed to reducing the negative consequences of adverse effects on patients' quality of life, which they mainly appraised through functionality (e.g., ability to work). However, among pharmacists, adverse effects affecting patients' quality of life were usually considered of lower priority than those impairing patients' physical health in the long term. Indeed, some adverse effects were mostly invisible but could lead to long‐term serious negative consequences, such as chronic diseases (e.g., kidney dysfunction), incapacities (e.g., because of a fall) or vital risks (e.g., respiratory arrest following an overdose). Pharmacists were particularly mindful of these long‐term serious adverse effects that they often found difficult to identify. When they prioritized prevention of irreversible harms over the quality of life, they reported facing some incomprehension from patients:

We pharmacists can sometimes get concerned about things that patients aren't all that worried about, such as kidney function or long‐term adverse effects. Patients don't necessarily see that aspect. What bothers patients, I think, is whatever affects their quality of life. (Pharmacist, Part.32‐FG8)

Nonetheless, adverse effects presenting risks for future health were also deemed concerning by many patients. The perspective of long‐term harms increased patients' uncertainty and powerlessness regarding their prognosis. Many felt they lacked information regarding such serious adverse effects. Some believed that pharmacists, physicians and/or pharmaceutical companies voluntarily hid harmful adverse effects, which highlighted their need to obtain clear information and be involved in their treatment choices:

I've done a lot of searching on the Internet and all I found were things from pharmaceutical companies saying you need to take this medication regardless of everything else, even if I end up ruining my liver. They really downplay the number of people who have serious side effects, like liver cancer, and all kinds of other side effects. (Patient, Part.18‐FG4)

#### 3.1.3 The dilemma between pain relief and adverse effect prevention

FG discussions suggested discrepancies between pharmacists' and patients' views of the importance given to pain versus potential serious harms. Seeking a balance between medication benefits and risks often entailed choosing between pain relief and adverse effect prevention. Many pharmacists insisted on their challenges in dealing with this ethical dilemma:

I have to take her [an older patient] off one of her pain medications, but then she starts to have pain again because of me. But if I don't remove it, she's going to fall and break her hip. That puts me in an awkward dilemma, it's a choice between having pain or falling and breaking something. (Pharmacist, Part.34‐FG9)

Several pharmacists explained that they could opt for dosage lowering or treatment cessation to limit adverse effects, even though the pain would remain unrelieved. For some of them, analgesics were not deemed ‘necessary’ medications because chronic pain was not a life‐threatening condition:

Any adverse effect can lead to stopping the treatment. The good thing, if you like, with pain medications, is that the treatment isn't necessary. For other conditions, heart problems, for instance, there are lots of preventive treatments [that are necessary]. When it comes to treating chronic pain, if the medication doesn't suit the patient, given that the purpose was to relieve pain, to improve quality of life, I have no problem taking them off it. (Pharmacist, Part.32‐FG8)

Some patients too preferred enduring more pain than facing harmful adverse effects. However, for many patients, the affirmation that chronic pain was not life‐threatening was not so evident. Several reported that pain could be so intense that it was hardly tolerable to live with it. They were thus willing to accept intense adverse effects as far as the pain would be effectively relieved:

It's not pleasant, but without them [painkillers], I wouldn't be here still. They're my crutch. I really don't have a choice at the moment. My medication is saving my life in a way, despite these problems of discomfort I have to put up with. I need this medication, regardless of the side effects. (Patient, Part.6‐FG2)

Finally, in many cases, the treatment failed to effectively relieve pain in addition to having adverse effects, leaving patients with a negative benefit/risk balance. Some patients questioned the relevance of taking pain medications under such conditions.

### 3.2 Managing adverse effects in the patient–pharmacist relationship

FG discussions underscored that the management of analgesic adverse effects took place both in patients' daily living activities and during interactions with prescribers and pharmacists, sometimes generating tensions in the decision‐making.

#### 3.2.1 Self‐management of the pharmacotherapy and adverse effects: Negotiating patients' expertise

To reduce adverse effects, several patients reported attempts to self‐manage their pharmacotherapy independently from healthcare providers. This included modifying the dosage or the moment of intake of a prescribed drug, as well as self‐medicating pain or adverse effects with over‐the‐counter and/or natural products. Several patients considered they had progressively acquired skills to manage their pharmacotherapy and better control the impact of adverse effects on their daily living. They claimed expertise for making decisions regarding their treatments (e.g., dosage adaptations). However, patients' experiential knowledge and self‐management practices were often subject to disagreements from physicians and pharmacists. Prescribers were often described as reluctant to involve patients in decisions regarding pain treatments:

I get told off for not taking my painkillers often enough. I try to discuss with my doctors, because you have to weigh the side effects against what you want to do. Yes, I should take it, but I can't follow the doctors' instructions because of the adverse effects, otherwise, I'd have to change jobs, there'd be more impact on my personal life. (Patient, Part.21‐FG5)

Many pharmacists, for their part, described the management of adverse effects as negotiations and patient education, rather than shared decisions. They were concerned by patients' other sources of information on their treatments (e.g., the internet, or patients' relatives). Several of them insisted on the importance of deconstructing patients' ‘beliefs’ regarding medications and tried to discourage patients' initiatives to modify dosages. Pharmacists were often worried about patients' self‐medication with natural medicine, as they thought patients often underestimated adverse effects and interactions with other drugs. They found it challenging to obtain information from patients regarding these practices and tried to educate them about potential harms while maintaining the dialogue:

What I explain to them is, let's say a given product contains 10 different natural products, then obviously with those 10 different things, there's definitely going to be some interactions with the 7 medications you're already taking. So, do what you like, but I'm not necessarily recommending that you take it, and if you do take it, and there's some reaction, then come back and see me again. (Pharmacist, Part.39‐FG10)

However, some patients described physicians and pharmacists accepting shared decision‐making. Having professional experience in the healthcare field was reported as a facilitator to obtain providers' recognizing of their experiential knowledge as patients:

I used to work in health care, and my anaesthetist at the pain clinic leaves me lots of latitude to manage my medication, so I have a major advantage in being able to self‐manage it. I'm the one who tells him: ‘Prescribe me more of that’ or, ‘I stopped taking that’. (Patient, Part.8‐FG2)

Other patients felt unsatisfied with the self‐management of their pharmacotherapy, especially when they perceived it as the result of insufficient involvement of providers rather than their own choice. These participants felt left alone with medication‐related risks and adverse effects, as for this patient treated with opioids:

My orthopaedist, when I last saw him, he said, ‘Do you need more?’ So, am I the one who decides? And the pharmacist suggests but doesn't impose anything. So, I'm the one who's managing my medication. I could have far more than I'm getting at the moment, but I think I'm already taking quite a lot. There's no one putting the brakes on for me. I'm the one making the decision, it's a little sad, but that's the way it is. (Patient, Part.6‐FG2)

#### 3.2.2 Managing opioid adverse effects: Pharmacist's policing practices

In the case of opioid treatments, pharmacists reported that their involvement in the management of adverse effects often took the form of monitoring and policing patients' medication use. Several patients using opioids for their chronic pain were concerned that pharmacists could suspect them of nonmedical use, and threaten the continuity of their treatment:

The first thing she [my new pharmacist] did was to report me to the College of Pharmacists, claiming I was a drug dealer. In her mind, it was impossible for someone to take so many drugs and still be able to walk and talk. (Patient, Part.23‐FG6)

Several pharmacists believed that the ‘police officer’ role was part of their missions with patients using opioids for chronic pain, though some of them disliked these tasks that were at the boundaries of healthcare:

As far as dependency goes and managing opioids, if you say to them, ‘No, I can't serve you today. You've still got some left at home’, then the person comes up with excuses. So, you might believe them the first time, but at some point, it's no longer acceptable. You have to set dates, to demand that people bring their medications into the pharmacy to see what they have left. All that kind of thing is really no fun. All that policing side of it, that can be really annoying sometimes. (Pharmacist, Part.41‐FG11)

Furthermore, opioid overdose prevention could cause challenging dialogue between patients and pharmacists. Naloxone, for example, was seen by several pharmacists as an important tool to reverse the effects of overdoses. However, patients facing opioid‐related stigma in their daily life were not likely to accept naloxone because they felt negatively labelled as opioid‐dependent or recreational consumers:

We pharmacists have the means to give out a kind of opioid EpiPen® called naloxone, so that patients can have it at home if they overdose on their opioids. So, if, for some reason, because they were in pain, they've taken a little more than they should have, and they have a breathing arrest, they can take naloxone. But getting patients to give this solution a try is a whole new challenge, because when you suggest it, they say, ‘I'm not a drug addict. I take medication to relieve my pain, but I'm not a junkie living on the street, so I don't think I need that’. It's really stigmatized. (Pharmacist, Part.38‐FG10)

#### 3.2.3 Healthcare and business running: Tension in the pharmacist's role

Many pharmacists considered they had, or should have, a key role to play in the management of analgesic adverse effects, including patient follow‐up, treatment optimization, patient education and support:

It's really important to follow up with the patient because some side effects are temporary and sometimes, just getting a bit of encouragement to keep going will help the patient to continue long enough to see the benefits. (Pharmacist, Part.30‐FG7)

Several patients viewed their pharmacist as an easily available expert on medications, more competent than physicians in addressing their problems with analgesic adverse effects:

Most of the time, I talk to my pharmacist because she knows more than the doctor about chronic pain. I learned from her that that there can be a serotonin effect between two medications that wouldn't normally be combined for the same patient. My doctor didn't know that much about it, and she didn't care that much either because we're talking about rare side effects, so I wasn't supposed to have them. But I did have side effects that were related to that. So, my pharmacist's expertise was better than my doctor's. (Patient, Part.18‐FG4)

However, other patients described pharmacists as medication retailers only. They did not recognize in them specific expertise. Several patients deplored that pharmacists were not likely to provide information on adverse effects unless patients took the initiative to ask. In addition, some reported having no discussions with pharmacists at all:

I have no relationship with pharmacists. I pick up my medication or have it delivered but I don't talk to anyone. I pay and that's it. The pharmacist's there to make sure the pills are okay but I have no relationship. I talk with the cashiers, with the technician who comes to fetch the prescriptions, that's all. (Patient, Part.3‐FG1)

Tensions between the role of community healthcare providers and the role of the business owners were omnipresent in pharmacists' discussions. Many of them explained that in an ideal world, they wished they could be highly involved in the management of adverse effects of chronic pain medications, through in‐depth one‐on‐one appointments with patients. However, many pharmacists described barriers related to insufficient time, lack of space and inappropriate remuneration policies in community pharmacies, which led them to prioritize fast medication delivery instead of close patient follow‐up:

If someone comes and makes an appointment, we're not compensated for that. At present, the system doesn't operate on a fee‐for‐service basis, but rather on the basis of the volume of renewed prescriptions, so profitability depends on volume. We're not paid for a pharmacotherapy follow‐up of a patient, that's where the problem lies. (Pharmacist, Part.37‐FG10)

For pharmacists working in family medicine groups, such constraints were less intense though time‐related barriers remained omnipresent.

## 4 DISCUSSION

The original contribution of this qualitative study was to provide an in‐depth understanding of the experience and management of analgesic adverse effects from the double perspective of chronic pain patients and community pharmacists. This study included all types of analgesic drugs while previous studies in the field focused on one single type of medication, usually opioids, and were mostly physician‐centred. Below, we discuss the study's most significant scientific contributions and practical implications regarding person‐centred care and shared decision‐making, and we suggest novel avenues to foster pharmacists' involvement in managing chronic pain pharmacotherapies and associated adverse effects.

This study showed that both patients and pharmacists constructed the benefit‐risk balance of adverse effects based on each patient's social and clinical characteristics. A given effect could be considered both an adverse effect by/for one patient or a beneficial effect by/for another patient. Participants highlighted the relative and variable nature of adverse effects according to patients' socioprofessional activities, age and comorbidities. This original finding testifies to participants' personalized approach to adverse effect prevention and management. This finding reinforces the relevance of person‐centred approaches to the management of pain in primary care.^3^, ^62^ The clinical appraisal of analgesic adverse effects needs to take place in partnership with patients, through the consideration of their preferences, as well as clinical, social and psychological dimensions.

In this study, the process of prioritizing adverse effects was not without its dilemmas for patients and pharmacists. Another qualitative study held in the general population described the benefit‐risk balancing of patients choosing between disease‐ and treatment‐related risks in the process of treatment initiation.^63^ A study among pain patients using opioids found that these patients often gave priority to pain relief even though they could aspire to quit opioids.^64^ Regarding providers, a study in the field of mental health highlighted their dilemmas in the face of treatment‐related harms, and their discursive strategies to value treatment benefits rather than adverse effects.^65^ Our findings underscored discrepancies between pharmacists' and patients' opinions regarding both the prioritization of adverse effects affecting the quality of life versus those presenting potential future harms, and the prioritization of adverse effect prevention versus pain relief. The mismatch between patients' and providers' priorities has been previously described as a major barrier to a satisfactory doctor‐patient relationship,^30^, ^66^, ^67^ including the management of opioid treatments for chronic pain.^38^, ^41^ The impacts of pain on patients' lives have been shown to be often underestimated in clinical settings, as the invisibility of pain can create misunderstandings and reduce providers' empathetic behaviours.^3^, ^64^, ^68^ It is essential to support pharmacists' practices with chronic pain patients through better training on pain assessment and recognition. Training initiatives promoting shared decision‐making could help tailor treatments to patients' expectations and needs while minimizing the risks associated with adverse effects.^38^, ^69^

With the view of fostering shared decision‐making, it is essential that pharmacists, and, more generally, health professionals, provide patients with information and support to make evidence‐based decisions about treatments and adverse effects.^63^ Our study highlighted some patients' willingness to be more involved in the management of their pharmacotherapy. Sociological research on the chronic illness experience showed that self‐management of medications (e.g., dosage adaptation) is often linked with patients' will to integrate the treatment in their daily life by reducing the impact of adverse effects on their activities.^47^, ^50^ A recent study described the use of over‐the‐counter medication as a harm reduction strategy enabling people with chronic pain to avoid the stigma associated with prescribed pain medication.^70^ Shared decision‐making could help reduce the potential harms associated with treatment self‐management, while recognizing patients' experiential knowledge and skills. Used in the mental health field, the ‘Gaining Autonomy in Medication’ (GAM)^71^, ^72^ approach could be a promising avenue in the area of chronic pain. This person‐centred approach based on patient empowerment provides guidance to foster collaborative management of medications by patients and providers,^73^ through the accessibility of information regarding medications, a personal reflection of patients' needs for quality of life and support to facilitate patient–provider communication.^71^ The GAM was shown to be efficient in reducing unsupervised treatment interruption and in fostering patients' understanding of their treatment.^71^ This approach could be useful to improve patient–pharmacist communication and foster shared decisions regarding chronic pain pharmacotherapies.

In the case of opioids, stigma played an important role in both patients' and pharmacists' experiences. Stigma experiences in various healthcare environments, and feelings of losing control of decisions regarding opioid therapies are frequent among patients treated with opioids for chronic pain, especially in the context of current opioid‐policy changes.^34^, ^38^, ^74^, ^75^ Pharmacists in this study could be suspicious regarding opioid nonmedical use and deploy policing practices. The social stigma surrounding opioids also hindered pharmacists' attempts to contribute to overdose prevention because patients did not feel targeted by prevention messages and thus often refused naloxone. Preventing opioid‐related risks at the pharmacy is critical during the current overdose epidemic. A global destigmatizing of opioids in the media and healthcare settings^38^, ^39^, ^56^ would be highly beneficial to prevent overdoses among chronic pain patients. Prevention campaigns providing accurate and nonjudgmental information would help patients better appraise the risks of overdose. It is also essential to educate and support pharmacists in adopting nonstigmatizing practices with their patients using opioids to maintain a positive relationship.

This study underscored the important role of pharmacists as easily accessible healthcare professionals for providing patients with information, monitoring and interventions regarding pain medications and adverse effects. Several other studies in the general population noted that pharmacists' availability was one major factor fostering patients' trust in their advice and the development of a sustainable relationship.^76^, ^77^ In Quebec, chronic pain patients' overall satisfaction regarding information received on their pharmacological treatment in primary care is low, especially in the case of patients experiencing adverse effects.^78^ Patients in our study had unequal access to reliable information about their treatment. Self‐management of adverse effects could be rendered more difficult to those who had less access to medical knowledge. Patients' narratives suggest that pharmacists would be first‐choice actors to provide expert information on chronic pain pharmacotherapies to improve the management of adverse effects. Many patients in this study would have welcomed a higher involvement of their pharmacist, especially those feeling left alone by providers. However, some patients considered that pharmacists were mostly medication sellers, therefore illegitimate to make treatment decisions. In another Canadian study, the lack of continuity in the relationship over time was pointed out as a factor leading patients to consider pharmacies as retail outlets rather than healthcare delivery facilities.^76^

Indeed, several pharmacists in our study reported healthcare system‐related barriers to their involvement in the management of analgesic adverse effects. Their remuneration system forced them to prioritize high volumes of medication delivery, thus hindering their ability to make in‐depth follow‐ups. A Canadian study underscored that financial factors were among the barriers preventing community pharmacists from taking part in the multidisciplinary treatment of chronic pain, as these pharmacists were dissuaded to seek nonpharmacological alternatives.^26^ As pharmacists' scope of practice has recently been extended to more involvement in treatment management in several jurisdictions, our study suggests that it will be essential to reform their remuneration policies and practice environment to enable them to spend more time doing patient follow‐up. This study demonstrated that pharmacists are willing to play a larger role in the management of adverse effects for chronic pain patients, which should encourage policymakers to support the alignment of their working conditions with the material requirements of this role.

Regarding study limitations, as shown in Table 2, most of the pharmacists who accepted to participate were relatively young, with less than 10 years of experience. The study may thus imperfectly capture the situation of more experienced pharmacists. However, some experienced pharmacists also provided their perspectives extensively, and we were particularly mindful of including them during the analysis. Furthermore, accessing the experience of younger pharmacists enabled us to suggest relevant improvements for current pharmacy training. In addition, this study was conducted in the Canadian province of Quebec, and it is important to acknowledge the potential influence of the local context in terms of pain management resources, healthcare system organization and regulation of the pharmacist profession. However, the situation of Quebec is similar to other provinces and countries, which makes our results potentially transferrable to geographic areas sharing similar characteristics. Results from this study could support further research in other countries to enrich our original findings through adaptations to diverse local contexts.

## 5 CONCLUSION

This study advanced knowledge by showing the complexity of defining, prioritizing and managing the adverse effects of chronic pain medications, as well as the dilemmas and discrepancies that adverse effect management can create in the patient–pharmacist relationship. With the vision of fostering patient‐centred management of adverse effects and shared decision‐making, it is essential to improve pharmacists' training in pain management, and to support them in delivering accurate information to their patients about analgesics and their adverse effects. Policy efforts need to be made to provide pharmacists with adequate material resources to play their critical role as primary care experts of pharmacological therapies.

## CONFLICT OF INTERESTS

The authors declare that there are no conflict of interests.

## ETHICS STATEMENT

The Research Ethics Committees of the *Centre Hospitalier de l'Université de Montréal* and *the Université du Québec en Abitibi‐Témiscamingue* have reviewed and approved this study (No. 19.367). All participants provided written informed consent to participate.

## AUTHOR CONTRIBUTIONS

The principal investigators in this study are Lise Dassieu and Emilie Paul‐Savoie. Lise Dassieu led the study, contributed to study design, recruitment, data collection supervision, data analysis and drafted the manuscript. Emilie Paul‐Savoie contributed to study lead, study design, recruitment and revised the manuscript. Elise Develay contributed to study administrative coordination, recruitment, data collection, data analysis and manuscript revisions. Ana Cecilia Villela Guilhon contributed to data collection, data analysis and manuscript revisions. Anaïs Lacasse and Line Guénette contributed to study design, recruitment and manuscript revisions. Kadija Perreault, Hélène Beaudry and Laurent Dupuis (person with lived experience) contributed to study design and manuscript revisions.

## Supporting information

Supporting information.

Supporting information.

## Data Availability

The data that support the findings of this study are available on request from the corresponding author. The data are not publicly available due to privacy or ethical restrictions.
