# Supplementary material for: Swallowing the pill of adverse effects: A qualitative study of patients' and pharmacists' experiences and decision‐making regarding the adverse effects of chronic pain medications
Source: Health Expect. 2021 Dec 21;25(1):394–407. doi: 10.1111/hex.13399 (PMC8849270; doi:10.1111/hex.13399)
Supplement: Supplementary file 1 — Supporting information. [file HEX-25-394-s001.pdf]

People living with chronic pain

Technical considerations

– Acknowledgements

– Specifics about how the session will proceed (length, confidentiality, roles of moderators, need to respect others' opinions, audio recording, compensation, etc.)

– Presentation of moderators: No conflict of interest. Sociologist/research professional and psychologist/doctoral student.

Introduction

– We are all here today to discuss the adverse effects of the medications you take for your chronic pain. An adverse effect is an unpleasant or harmful side effect, or an illness, that occurs after taking medication (suspected or established cause).

– What the concept of adverse effect actually covers is left to your interpretation. We won't be examining a pre-established list of adverse effects, but rather exploring what effects you identify as being adverse, and why they are adverse for you.

Questions

1) To start off, I'd like to hear what you have to say about the adverse effects of painkillers you've tried, based on your personal experience.

2) What adverse effects are you the **most concerned about**? And why?

3) How (when, in what contexts) do these adverse effects interfere with your **day-to-day life**? And why?
   Prompts: How do these adverse effects interfere with your professional life? And with your personal life?

4) What do you do on a day-to-day basis to **manage** these adverse effects?
   Prompts: Do you sometimes adapt the times/dosages of your medication depending on what you have to do during the day (ask for examples of situations)? Have you ever stopped taking your medication (even temporarily) so as to control the adverse effects?

5) What kind of **relationship do you have with pharmacists** regarding the adverse effects of painkillers?
   Prompts: What are the positive and the negative aspects of this relationship? Do your pharmacists listen to you? What advice do they give you? Have you ever had any disagreements? If so, what about?

6) Do your painkillers have any **positive side effects**, in your view? If so, what are they? In what way are they positive?

7) If you had the power to create **the ideal medication** for your chronic pain, what side effects would you do away with and which ones would you keep, and why?
