# Supplementary material for: Swallowing the pill of adverse effects: A qualitative study of patients' and pharmacists' experiences and decision‐making regarding the adverse effects of chronic pain medications
Source: Health Expect. 2021 Dec 21;25(1):394–407. doi: 10.1111/hex.13399 (PMC8849270; doi:10.1111/hex.13399)
Supplement: Supplementary file 2 — Supporting information. [file HEX-25-394-s002.pdf]

Pharmacists

Technical considerations

– Acknowledgements

– Specifics about how the session will proceed (length, confidentiality, roles of moderators, need to respect others' opinions, audio recording, compensation, etc.)

– Presentation of moderators: No conflict of interest. Sociologist/research professional and psychologist/doctoral student.

Introduction

– We are all here today to discuss the adverse effects of chronic pain medications. An adverse effect is an unpleasant or harmful side effect, or an illness, that occurs after taking medication (suspected or established cause).

– What the concept of adverse effect actually covers is left to your interpretation. We won't be examining a pre-established list of adverse effects, but rather exploring what effects you and your chronic pain patients identify as being adverse, and why they are adverse. We're interested in the pharmacist's role in managing adverse effects, identifying what the adverse effects are and why they are considered to be adverse. If you can give examples from your practice, it will be very helpful.

Questions

1) What **challenges** do you face in managing the adverse effects of the medications taken by your patients for chronic pain?

2) What adverse effects of painkillers are you **the most concerned about?** And why?
Prompts: What adverse effects are the most challenging for you in terms of patient care? Why?
What are your main concerns with respect to polypharmacy or polymedication (use of several medications at the same time)? With respect to over-the-counter or natural medications, including cannabis?

3) What do you do to **manage** the adverse effects reported by your patients? And to manage associated risks/consequences?
Prompts: Do you act differently depending on the class of drug? Depending on the patient? Depending on the type of adverse effect? And if so, why?
What challenges do you face? Opioids?

4) In your view, what adverse effects are your patients **the most concerned about**? Why?
Prompts: How do these effects differ from the adverse effects you are most concerned about? What differences are there between classes of drugs?

5) Based on your experience, what adverse effects could **lead to stopping pharmacological treatment?** And what would the reasons be?
Prompts: What do you do to help your chronic pain patients get off a medication? (and in the specific case of people trying to get off opioids) Who is asking to get off the drug?

6) Overall, how would you describe your **relationship with your chronic pain patients** who suffer adverse effects?
Prompts: What are the positive and the negative aspects of this relationship? How do patients react to your advice? Have you ever had relationship problems or disagreements with your patients? About what? (please give concrete examples, such as the last time that happened to you ...)

7) If you had the power to create **the ideal medication** for your patients living with chronic pain, what side effects would you do away with and which ones would you keep, and why?

8) What **changes** would you like to make so that you could provide better care to your chronic pain patients in a pharmacy setting? (*Recommendations*)
